# Is Creative Description Always Effective in Purchase Intention? The Construal Level Theory as a Moderating Effect

**DOI:** 10.3389/fpsyg.2021.619340

**Published:** 2021-07-05

**Authors:** Fei-Si Yao, Jing-Bo Shao, He Zhang

**Affiliations:** Department of Economics and Trade, School of Management, Harbin Institute of Technology, Harbin, China

**Keywords:** text description style, creative description, construal level, purchase intention, moderating effect

## Abstract

Recent years has witnessed a rapid growth in online shopping. This paper draws from the construal level theory to examine the divergent effects of the creative text descriptions of products on consumers' purchase intention in an online context. It also investigates consumers' construal level and the moderating role of construal level in this relationship. An assumption has been made that the creative description embraces more rhetorical devices with analogies. In doing so, such texts are in need of consumers who are having a more abstract, top-down, flexible mindset, which makes it more persuasive to some consumers with high-level construal. Three experiments add evidence to this study. These results suggest that the creative text descriptions are generally more persuasive than the non-creative ones in an online context, and that the persuasiveness of the creative descriptions can be accentuated (vs. attenuated) especially for high- (vs. low-) level construal individuals. The findings hold various theoretical implications for the creative marketing messages and construal level theory. First, in the current research, broadening, and integrating relevant research were possible by exploring the creative language in an online context. Also, it demonstrates that construal level—that is, consumers' internal thoughts, rather than external factors—influences their preference for a creative description style, thus helping extend the applications of the construal level theory to the field of creative marketing communications and integrate the research discoveries in metaphor communication.

## Introduction

Improved logistics chains and the popularization of “smart” mobile devices have given consumers access to online shopping platforms anytime and anywhere when they choose to shop (Batra and Keller, 2016). These changes motivate online retailers to compose appealing and persuasive messages and advertisements. To attract consumers' attention and imbue a product with an appealing aura, many online retailers use creative text to describe products [i.e., the text descriptions that include more rhetorical devices (Ang and Low, 2000; Dahlén et al., 2008; West et al., 2019)]. At first glance, the creative text descriptions appear to be intuitively more persuasive than the non-creative ones (i.e., the text descriptions that convey plain dictionary meaning and do not include rhetorical devices McQuarrie and Mick, 1999, 2003; Tom and Eves, 1999; McQuarrie and Phillips, 2005; Phillips and McQuarrie, 2009; Kronrod and Danziger, 2013; Dahlén et al., 2018). However, scholars question the advantages of a creative description, and the lack of consensus regarding which description is better: creative or non-creative (Friestad and Wright, 1994; Kover et al., 1995, 1997; Campbell and Kirmani, 2000; Mothersbaugh et al., 2002; Xu and Wyer, 2010; West et al., 2019).

Previous research has explored the boundary conditions from the perspective of consumers' ability (e.g., whether the advertising messages are presented incidentally Phillips and McQuarrie, 2009) and motivation (e.g., consumers' ability to process metaphor language Phillips and McQuarrie, 2009) and consumers' differences in need for cognition Chang and Yen, 2013). However, consumers' ability and motivation to process advertising materials may have greatly weakened in today's increasingly digitized and multimedia environment (West et al., 2019), since attention to the contemporary information era is precious. In other words, the factors explored in the literature may not necessarily apply to this study, and more internal factors of consumers could provide a new perspective. Fortunately, from the perspective of consumers themselves, West et al. (2019) propose that an individual's information process or a cognitive mindset may play a critical role in improving the persuasiveness of creative messages. In terms of the perspective, relevant research reveals that consumers may prefer to process either verbal or visual information (Ko-Januchta et al., 2017). Similar and related to this cognitive style is the construal level theory, which focused on an individual's mindset on different construal levels, regardless of verbal or visual information. Moreover, research on metaphors, which are highly related to creativity (Marin et al., 2014; Sang and Yeo, 2019), has revealed that an individual's construal mindset is linked to the processing and response to the communications with metaphors (Landau et al., 2019; Sang and Yeo, 2019). Moreover, there also exists evidence that “top-down” processing (Zinken and Zinken, 2007; Gibbs, 2013) and flexible cognition (Förster et al., 2004), which are embedded in high-level construal, are beneficial in interpreting rhetoric devices. Therefore, this study identifies consumers' construal level as a moderating factor to provide new insights into the boundary conditions of creative text's persuasiveness.

The current research gains insights from research on the construal level theory (Trope et al., 2007; Förster, 2009), and aims to extend the current understanding of the effectiveness of creative text descriptions by investigating the divergent effects of different text description styles on consumers' purchase intention and considering the moderating role of consumers' construal level in an online context. In this study, first, we posit two hypotheses and develop a way to model construal level's moderating effect on text's persuasiveness *vis-à-vis* purchase intention. We then perform three experiments to test the model and hypotheses. We conclude by reflecting on our findings. This research provides two primary theoretical contributions. First, it is a broadened and integrated one of relevant research by exploring the employment of creative language in an online context. Second, it provides a boundary condition for the effectiveness of a creative text description by identifying the construal level as a moderator, thus helping extend the previous research in traditional creative advertising and integrate the research discoveries in metaphor communication.

## Theory and Hypotheses

### Text Description Style and Purchase Intention

The literature identifies creative and non-creative styles as the two mainstream approaches to describe a product in the text (Kronrod and Danziger, 2013). The former uses more rhetorical devices with figurative language using analogies, such as metaphors and personification (Ang and Low, 2000; Dahlén et al., 2008; Landau et al., 2019; West et al., 2019; Yao and Shao, 2021). The non-creative text descriptions are just what their name suggests—they do not contain rhetorical appeals, and the words and phrases have plain dictionary meaning (Kronrod and Danziger, 2013; West et al., 2019; Yao and Shao, 2021). For example, the description of earphones as “Soft and skin-friendly silicone earplugs, your ears feel like they are touching a baby's skin” from Netease YANXUAN is more creative than the non-creative statement “Featuring a comfortable silicone earplug design” from Taobao (see the Supplementary Material for more marketing practices for the two description styles). In the current research, we concentrate on the persuasiveness of the creative text descriptions, and the non-creative text descriptions are used for a comparison.

In advertising, creative language is generally and intuitively believed to be more persuasive than the non-creative language (Aaker, 1975; McQuarrie and Mick, 1999, 2003; McQuarrie and Phillips, 2005; Phillips and McQuarrie, 2009; Chang and Yen, 2013; West et al., 2019). Research on metaphor advertising, a specific category of creative advertising, provides abundant evidence that such creative marketing messages are more eye-catching and appealing, and that when consumers are pushed to actively engage in creative messages, they appreciate their artfulness (Harris et al., 1999; Sopory and Dillard, 2002; Phillips and McQuarrie, 2009) and feel more positively about the product or brand (McQuarrie and Mick, 1999, 2003; McQuarrie and Phillips, 2005; Phillips and McQuarrie, 2009; Dahlén et al., 2018; West et al., 2019). Studies have shown that consumers see the product messages as “the literature of economic change” (Scott, 1994, p. 464), designed to persuade them (Hansen and Scott, 1976; Coleman, 1990), and thus they expect marketing messages to be amusing, creative, and artful (Nilsen, 1976; Wyckham, 1984; Stern, 1988). Moreover, highly creative message presentations have been shown to increase a message's persuasiveness by provoking a deeper thought (Mothersbaugh et al., 2002) and more agreement (Mcguire, 2000) in consumers. Given the literature, we presume that the creative text descriptions are generally more persuasive than the non-creative ones and that they have predictable and positive impacts on consumers' purchase intention. Thus, our first hypothesis is as follows.

*H1: Creative text description* is *more persuading in promoting consumers' purchase intention than noncreative text description*.

### The Moderating Role of Construal Level

The effects of the creative text descriptions are not, however, straightforward. Scholars have challenged the presumed positive effect of a creative text description by proving that using creative language is not necessarily more persuasive as doing so does not add to the message's functionality (Kover et al., 1995, 1997). Other studies indicate that consumers consider marketers' use of exaggerated, intense rhetorical devices to be the norm (Friestad and Wright, 1994; Kronrod and Danziger, 2013) and are, hence, largely immune to their effects (Friestad and Wright, 1994; Campbell and Kirmani, 2000; Xu and Wyer, 2010). To better understand the role of creative language, some studies have investigated consumers' agency by considering whether the advertising messages are presented incidentally (Phillips and McQuarrie, 2009), by assessing consumers' ability to process metaphor language (Phillips and McQuarrie, 2009), or by exploring consumers' differences in need for cognition (Chang and Yen, 2013). However, those previous researches are based on the traditional advertising context, and an individual's information process or a cognitive mindset may be more critical and effective in today's digitized and multimedia environment (West et al., 2019). Moreover, rhetoric devices and construal level are positively related in metaphor communication (Shan et al., 2017; Landau et al., 2019). Therefore, we identify the boundary condition from the perspective of the construal level.

The construal level theory (Trope et al., 2007) assumes that people process information at either a high or a low level of construal. High-level construal mindsets are abstract, “top-down,” decontextualized, and superordinate (Vallacher and Wegner, 1989; Trope et al., 2007). In contrast, low-level construal mindsets are concrete, “bottom-up,” contextualized, and subordinate (Vallacher and Wegner, 1989; Trope et al., 2007). Scholars have indicated that construal level is not only a personal chronicle trait but also a situational factor, which can be affected by context cues such as psychological distance (Vallacher and Wegner, 1989; Trope et al., 2007). Psychological distance defined as the extent of deviation from direct experience in time, space, social distance, or a hypothetical state influences people's responses by altering construal level and how they interpret information (Trope et al., 2007). Individuals tend to use a high-level construal and abstract mindset when evaluating psychologically distant events, and a low-level construal and concrete mindset for psychologically proximate activities.

This study predicts that consumers' construal level could moderate the relationship between the style of a product's descriptive text and consumers' purchase intention for the following reasons. Interpreting the creative text descriptions require consumers to demonstrate a high level of construal. For example, metaphor is a special form of the general category of creative language and is most investigated in marketing communications. Recent research has found that abstract mindsets help recipients to process metaphors in health communication (Landau et al., 2019). A creative text description in this research contains more rhetoric devices with figurative language using analogies such as metaphors and personification (Ang and Low, 2000; Dahlén et al., 2008; West et al., 2019), which means claiming product's key benefits with “remote conveyors” that are seemingly unrelated to the product (Althuizen, 2017). More precisely, for example, in the Supplementary Material, “female treasure” has been used to depict the shea butter's superior advantage while the two objects have different surface attributes. Therefore, to better understand the product merits, one needs to step back and look beyond the concepts' concrete details, which in turn capture how they share an underlying structure (Landau et al., 2019).

In other words, the use of rhetorical devices in marketing requires consumers to make abstract, creative, and flexible connections between what a product promises and what it actually is (Lakoff and Johnson, 1980; McQuarrie and Mick, 2003; Förster et al., 2004; Vervaeke and Kennedy, 2004; McQuarrie and Phillips, 2005; Zinken and Zinken, 2007; Landau et al., 2010; Gibbs, 2013; Yang et al., 2019). Also, other relevant studies have indicated that individuals' processing of rhetoric devices is related to various elements of their mindset, such as “top-down” thinking (Gibbs, 2013) and cognitive flexibility (West et al., 2019)—both of which are related to high-level construal. In summary, individuals who are generally oriented to use an abstract mindset will be more likely to appreciate how one conveyer provides a useful framework for understanding another, even though they look unrelated (Landau et al., 2019). They are prone to interpret a creative marketing message in ways that correspond to the provided online retailer's source (Förster et al., 2004; Kille et al., 2017; Landau et al., 2019), which might, in turn, enhance their purchase intention. Consumers with low construal levels have concrete mindsets, and they are more likely to concentrate on the specific details between the “remote conveyer” and the product attributes. Thus, they would overlook their shared structure (Landau et al., 2019) because of the less creative and flexible processing ways (Förster et al., 2004; Kille et al., 2017). As a result, they may not be as persuaded by the creative text descriptions of products as their high-level construal counterparts.

From the above logic, we formed our second hypothesis:

*H2: Construal level moderates the relationship between text description style and consumers' purchase intention. Specifically, the purchase intention of high-level construal consumers is more effectively persuaded by creative text description than that of low-level construal consumers*.

**Figure 1:**
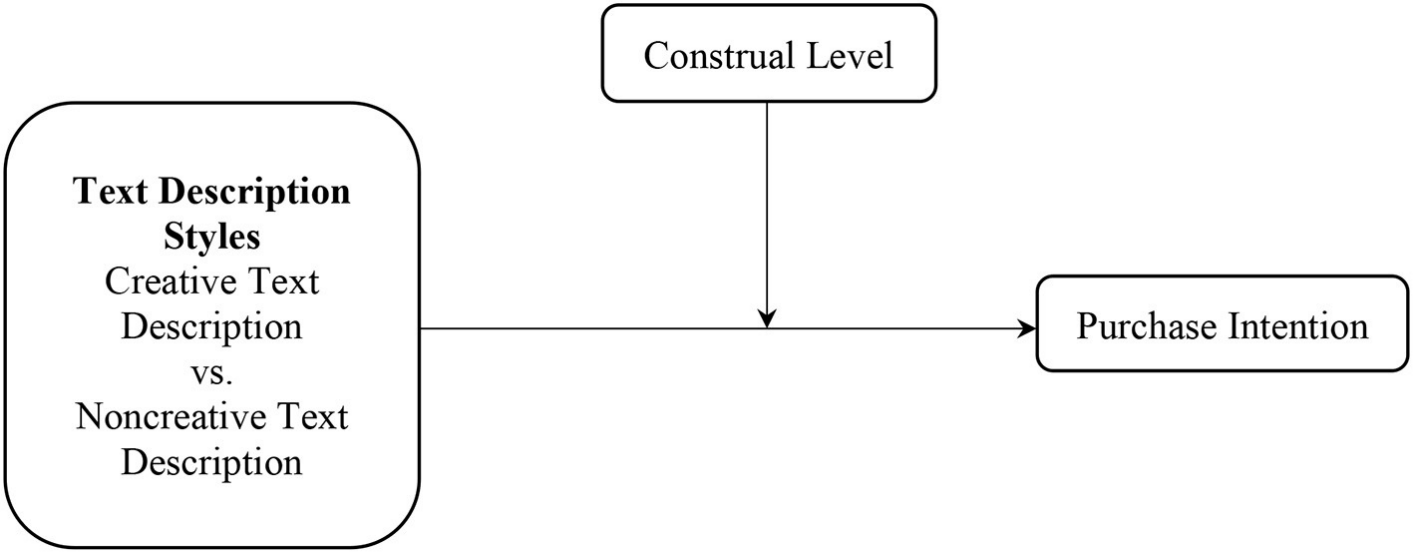
Theoretical model.

Combining H1 and H2, the theoretical model for the current research is presented in Figure 1.

## Testing of Hypotheses and the Moderate Model

### Study Summary

We tested our hypotheses using three experiments (Studies 1–3). Each experiment employed different study designs and operationalized construal level in a different manner to demonstrate the validity and robustness of our framework. Studies 1 and 2 employed a between-subject study design, and Study 3 used a between- and within-subject mixed study design. Study 1 manipulated consumers' construal level through category and exemplar tasks (Fujita et al., 2006; Wang et al., 2017), Study 2 measured consumers' chronic construal level (Wang et al., 2017), and Study 3 operationalized consumers' construal level through social distance to further validate the moderating role of construal level (Baskin et al., 2014; Goodman and Lim, 2018).

Online product descriptions typically have three main elements: pictures, the product's title or brand name, and a text description of the product. In our manipulation study, we kept the pictures and brand names constant across each experiment and varied the text description and title (except the brand) to communicate the same message in either a creative (employing more rhetorical figures) or a non-creative (without rhetorical figures) way, based on the research of Dahlén et al. (2008) and Kronrod and Danziger (2013). The contents of the text descriptions were adapted from JD.COM, NetEase YANXUAN, and Taobao to enhance their external validity. The non-creative descriptions were largely based on the creative descriptions to ensure that their core content corresponded to one another (see the Supplementary Material for a complete product's text descriptions). Three different products were selected for each study. To avoid extra-experimental artifacts from using an existing brand, we fabricated the brand or e-commerce platforms employed in the experimental instruction text (Laczniak et al., 1999).

Since the creative text descriptions contained more rhetoric devices and the length of descriptions of two styles was not completely consistent, we conducted three separate pretests, one before each study, to ensure that our manipulation of the text descriptions had the same perceptions of message content and to rule out the concerns that participants' cognitive load of the two groups' descriptions differ from each other. The details of all three pretests are presented as a Supplementary Material.

All employed scales and questions were presented in Chinese. To ensure the validity of their translation, we had one bilingual professor to translate the original scales into Chinese, and then we had these scales back-translated into English by two bilingual PhD students (Brislin, 1980). All translators assessed whether the scales had the same meanings, and the inconsistencies were smoothed over by a consensus (Brislin, 1980).

### Study 1

To provide an initial support for our hypotheses, Study 1 tested whether the creative text descriptions influence consumers' purchase intentions (H1) and whether the high-level construal consumers are more persuaded by the creative text descriptions (H2). With this purpose, we carried out a between-subject study design by priming construal level through a situational cue that manipulated consumers' construal levels through category and exemplar tasks (Fujita et al., 2006; Wang et al., 2017) and presenting the creative and non-creative descriptions of products for different groups.

#### Method

##### Participants

A total of 727 Chinese participants (273 males and 454 females) were recruited from WJX.cn for an online experiment. They received a small monetary reward for their time. The majority (68.1%) were aged 26–40 years. The sample sizes were based on *a priori* power analysis and we sought a sample size, which is large enough to detect the mean effects reported in social psychology (*r* = 0.21 with 85% power at α = 0.05; Richard et al., 2003). The study employed a 2 (text description style: creative and non-creative) × 2 (construal level: high and low) between-subjects design. Participants were randomly assigned to one of those four conditions.

##### Construal Level Manipulation

Study 1 primed construal level by situational factors and followed the manipulation procedure and manipulation check of Hong and Lee (2010). In Hong and Lee's (2010) research, the construal level manipulation was based on the methods employed by Fujita et al. (2006). Specifically, 15 nouns (e.g., flower) were presented to the participants in the beginning. Those, in the high-level construal condition, were asked to generate a superordinate category label for each noun (e.g., plant), and those in the low-level construal condition were asked to generate a subordinate exemplar (e.g., camellia). The manipulation check items would be demonstrated in more detail later.

##### Shopping Scenario

Participants then proceeded to an experimental shopping scenario. They were asked to imagine that with the accelerating life pace, various modern diseases (such as lower back pain and cervical pain) also follow; therefore, they were searching online for a massager to relieve pains and pressure. Then, they encountered the picture and the text description of a massager with a fabricated brand name Juno. The text description's style was manipulated as mentioned above.

##### Measurement

After reading the product description, participants indicated how likely they were to purchase the massager and rated the manipulation check questions for text description style and construal level.

Based on the research by Holzwarth et al. (2006) and Visentin et al. (2019), purchase intention was measured in participants' responses to the three questions on a 7-item Likert scale, where 1 = completely disagree and 7 = completely agree (see the details in Table 1). The average score of their responses was the single purchase intention score (α = 0.73).

**Table 1:** Constructs and measurements used in this research.

**Study ID**	**Construct (Number of items; Source)**	**Items**
Study 1	Purchase Intention (3; Holzwarth et al., 2006; Visentin et al., 2019)	1. I can imagine buying a massager from this brand. 2. The next time I buy a massager, I will take this brand into consideration. 3. I am very interested in buying a massager from this brand.
	Manipulation Check for Text Description Style (3; adapted from Madrigal and King, 2017)	1. How do you rate the creativity of the language used in the product's text description? 2. How do you rate the interestingness of the language used in the product's text description? 3. How do you rate the novelty of the language used in the product's text description?
	Manipulation Check for Construal Level (1; adapted from Hong and Lee, 2010)	1. Please imagine that you have decided to purchase a massager and indicate when you would buy the massager.
Study 2	BIF as Manipulation Check for Construal Level (25; Vallacher and Wegner, 1989)	1. Making a list: a. Getting organized^a^ b. Writing things down 2. Reading a. Following lines of print b. Gaining knowledge^a^ 3. Joining the Army a. Helping the Nation's defense^a^ b. Signing up 4. Washing clothes a. Removing odors from clothes^a^ b. Putting clothes into the machine 5. Picking an apple a. Getting something to eat^a^ b. Pulling an apple off a branch 6. Chopping down a tree a. Wielding an ax b. Getting firewood^a^ 7. Measuring a room for carpeting a. Getting ready to remodel^a^ b. Using a yardstick 8. Cleaning the house a. Showing one's cleanliness^a^ b. Vacuuming the floor 9. Painting a room a. Applying brush strokes b. Making the room look fresh^a^ 10. Paying the rent a. Maintaining a place to live^a^ b. Writing a check 11. Caring for houseplants a. Watering plants b. Making the room look nice^a^ 12. Locking a door a. Putting a key in the lock b. Securing the house^a^ 13. Voting a. Influencing the election^a^ b. Marking a ballot 14. Climbing a tree a. Getting a good view^a^ b. Holding on to branches 15. Filling out a personality test a. Answering questions b. Revealing what you're like^a^ 16. Toothbrushing a. Preventing tooth decay^a^ b. Moving a brush around in one's mouth 17. Taking a test a. Answering questions b. Showing one's knowledge^a^ 18. Greeting someone a. Saying hello
		b. Showing friendliness^a^ 19. Resisting temptation a. Saying “no” b. Showing moral courage^a^ 20. Eating a. Getting nutrition^a^ b. Chewing and swallowing 21. Growing a garden a. Planting seeds b. Getting fresh vegetables^a^ 22. Traveling by car a. Following a map b. Seeing countryside^a^ 23. Having a cavity filled a. Protecting your teeth^a^ b. Going to the dentist 24. Talking to a child a. Teaching a child something^a^ b. Using simple words 25. Pushing a doorbell a. Moving a finger b. Seeing if someone's home^a^
	Purchase Intention (1; adapted from Baskin et al., 2014)	1. Please imagine to choose between the two e-commerce platforms and indicate your relative purchase intention.
Study 3	BIF (25; Vallacher and Wegner, 1989)	The same items used in study 2.
	Manipulation Check for Text Description Style (3; adapted from Madrigal and King, 2017)	The same item used in study 1.
	Purchase Intention (2; Burgers et al., 2015)	1. Whether it is likely that you would buy the hand cream. 2. Whether it is likely that you would recommend buying the hand cream to a good friend.

a *represents the high construal level option*.

To check the effectiveness of our manipulation for the text description style, participants were required to rate the description's creativity on three items (α = 0.77; Madrigal and King, 2017) on a 7-point scale anchored by 1 = not at all and 7 = very much (see the details in Table 1).

To check the manipulation for construal level, participants were asked to imagine that they had decided to purchase a massager and indicate when they would buy the massager on a 13-point scale (where 1 = today and 13 = after 6 months). This measurement was adapted from Hong and Lee (2010) because previous research indicated that high-level construal makes people to think that they would perform actions in a more distant future (Liberman et al., 2007). Finally, participants gave their demographic information (gender, age, the level of education completed, etc.).

#### Results

##### Manipulation Check

As predicted, the category and exemplar task got affected when participants thought that they might buy the product: *M*_highCL_ = 8.93, SD = 2.83 vs. *M*_lowCL_ = 8.06, SD = 2.77, *F*_(1, 725)_ = 17.70, *p* = 0.000, η^2^_*p*_ = 0.02. Participants judged the creative description being significantly more creative than the non-creative description [*M*_creative_ = 5.32 vs. *M*_non−creative_ = 4.90, SD = 1.00, *F*_(1, 725)_ = 20.15, *p* = 0.000, $\eta_{\text{p}}^{2}$ = 0.03].

##### Purchase Intention

**Figure 2:**
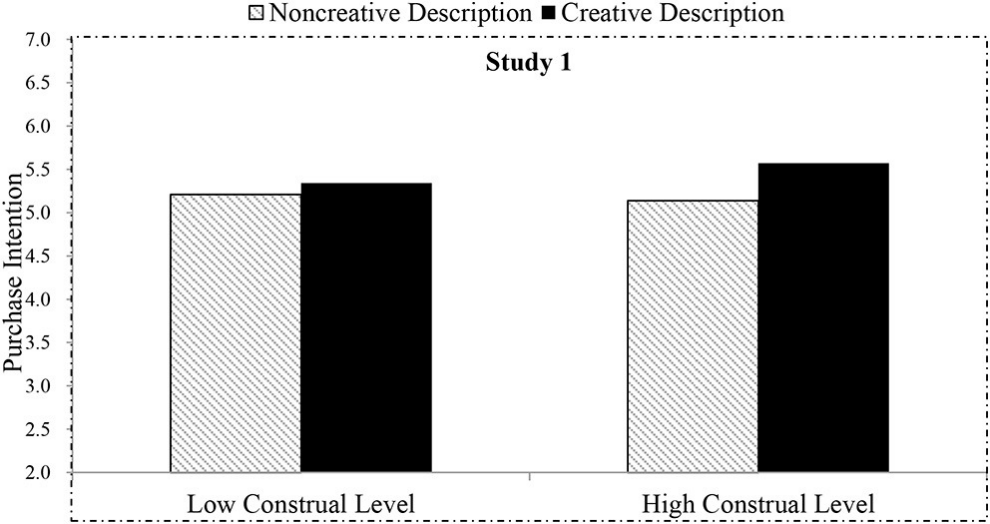
Effect of text description style and construal level on purchase intention in Study 1.

Being consistent with H1, our one-way ANOVA analysis using gender, age, and education as control variables showed that the text description style had a significant effect on participants' purchase intention [*M*_creative/purchaseintention_ = 5.45, SD = 0.83 vs. *M*_non−creative/purchaseintention_ = 5.17, SD = 0.96, *F*_(1, #722)_ = 16.55, *p* = 0.000, η^2^_*p*_ = 0.02]. The predicted interaction between the description style and construal level emerged [*F*_(1, s720)_ = 5.03, *p* = 0.025, η^2^_*p*_ = 0.007; see Figure 2], confirming H2. Specifically, among the participants in the high construal level group, those in the creative description condition were more likely to purchase the presented products than their counterparts in the non-creative description group [*M*_creative/purchaseintention_ = 5.57 vs. *M*_non−creative/purchaseintention_ = 5.14, SD = 0.86, *F*_(1, 360)_ = 20.55, *p* = 0.000, η^2^_*p*_ = 0.05]. However, we did not find a significant reverse effect for a low-level construal group. Indeed, the participants in both creative and non-creative description conditions reported to have almost an equal-purchase intention [*M*_creative/purchaseintention_ = 5.34 vs. *M*_non−creative/purchaseintention_ = 5.21, *F*_(1, 357)_ = 2.05, *p* = 0.153, η^2^_*p*_ = 0.01].

#### Discussion

By manipulating the construal level, Study 1 provides initial evidence for our hypotheses: the creative product description in an online context has persuasive advantages on purchase intention (H1) and an individual's high construal level accentuates the persuasion (H2). The results of Study 1 in the context of online shopping are consistent with the relevant research that confirms that the creative marketing message has general advantages (West et al., 2019) and high-level construal could enhance the effectiveness (Landau et al., 2019).

However, except for a situational prime, an individual's construal level could also be internal and chronical (Vallacher and Wegner, 1989). To further guarantee the robustness of our framework, we conducted Study 2 to directly measure individual chronic construal level without manipulation.

### Study 2

Study 2 intended to replicate the results of Study 1 and provide further evidence for our framework. Rather than using a situational prime, Study 2 considered construal level as a personal trait and directly measured participants' chronic tendency to construe the information in a high or low level (Wang et al., 2017). Thus, it employed a construal level (measured) by the text description style (creative vs. non-creative) of the between-subjects design.

#### Method

##### Participants

We recruited 383 Chinese participants (131 males and 252 females) from WJX.cn in exchange for payment. The majority of them (66.9%) were aged 26–40 years. The sample sizes were based on *a priori* power analysis, and we sought a sample size, which is large enough to detect the mean effects reported in social psychology (*r* = 0.21 with 85% power at α = 0.05 Richard et al., 2003). They were randomly assigned to one of the following two experimental conditions.

##### Procedure

As we treated construal level as personal traits, similar to the study procedure by Wang et al. (2017), the participants were required to complete a behavior identification form (BIF; Vallacher and Wegner, 1989; see the Supplementary Material for complete scale) in the beginning of the experiment. The BIF is a 25-item questionnaire to measure an individual's differences in action identification. Each item indicates a target behavior (e.g., locking a door) and requires the participants to choose between a high-level (e.g., securing the house) or a concrete, low-level representation of that action (e.g., putting a key in the lock). Preferences for low- and high-level representations were coded as 0 and 1, respectively. Participants' scores for each of the 25 items were added up to yield their overall BIF score (α = 0.77; see the details in Table 1). Higher BIF scores indicated a greater tendency toward high-level construal. This was treated as a measurement of their chronic construal levels.

Then, the participants proceeded to the shopping scenario. The improved living standards have increased the demand for hand and body care. Therefore, we chose hand cream as a focal product. Participants were asked to imagine that they wanted to buy a hand cream and were searching for it online. They were then presented with the picture and the text description of a hand cream with a fabricated brand name Hestia. The text's description style was manipulated depending on the participants' condition.

##### Measurement

Participants indicated their purchase intention and completed the manipulation check question and demographic measures. Purchase intention was measured by using two items (α = 0.72; Burgers et al., 2015; see the details in Table 1). The manipulation check question for the text description (α = 0.81; Madrigal and King, 2017) and demographic measures were similar to that in Study 1.

#### Results

##### Manipulation Check

The participants of the creative description group judged the used language to be more creative than those of the non-creative description group [*M*_creative_ = 5.40, SD = 1.00 vs. *M*_non−creative_ = 4.88, SD = 1.12, *F*_(1, 381)_ = 23.67, *p* = 0.000, η^2^_*p*_ = 0.06]. This demonstrated an effective manipulation of the text descriptions.

##### Purchase Intention

We employed the traditional hierarchical regression method to conduct a preliminary analysis of the data since the construal level in Study 2 was a continuous variable as we directly measured it. Through the hierarchical regression, we constructed the interaction term of an independent (text description style) and a moderating variable (construal level) to investigate a moderating effect.

Table 2 displays the correlations between all measured variables. Being consistent with our predictions, the hierarchical regression results indicated that the creative description exerted a significant positive effect on purchase intention (*B* = 0.29, SE = 0.11; *p* = 0.011; see the details in Table 3), confirming H1. In addition, being consistent with H2, the text description style and construal level had a significant interaction effect on participants' purchase intention (*B* = 0.07, SE = 0.02; *p* = 0.003; see details in Table 3).

**Table 2:** Descriptive statistics and correlations in Study 2.

	**1**	**2**	**3**	**4**	**5**	**6**
1 ^a^Gender	–					
2 ^b^Age	−0.20^**^	–				
3 ^c^Education	0.07	0.21^**^	–			
4 Text description style	0.00	0.06	0.05	–		
5 Construal level	−0.13^*^	0.05	−0.05	0.07	–	
6 Purchase intention	0.08	0.09	0.05	0.15^**^	0.14^**^	–
Mean	–	–	–	0.52	15.45	5.31
S.D.	–	–	–	0.50	4.64	1.11

*N = 383*,

* *p < 0.05*,

** *p < 0.01*.

a *Gender (“0” male; “1” female)*.

b *Age (“1” under 18; “2” 18–25; “3” 26–30; “4” 31–40; “5” 41–50; “6” 51–60; “7” above 60)*.

c *Education (“1” high school and blow; “2” professional training; “3” bachelor; “4” master; “5” doctor and above)*.

**Table 3:** The results of hierarchical regression in Study 2.

**Variables**	**Purchase intention**		
	**Model 1**	**Model 2**	**Model 3**
^a^Gender	0.24 (0.12)	0.27 (0.12)^*^	0.27 (0.12)^*^
^b^Age	0.11 (0.06)	0.10 (0.06)^+^	0.10 (0.06) ^+^
^c^Education	0.05 (0.10)	0.05 (0.10)	0.04 (0.10)
Text description style		0.29 (0.11)^*^	−0.80 (0.38)^*^
Construal level		0.03 (0.01)^**^	−0.00 (0.02)
Text description style × Construal level			0.07 (0.02)^**^
*R*^2^	0.02	0.06	0.08
Adjusted *R*^2^	0.01	0.05	0.06
Δ*R*^2^	0.02	0.04	0.02

*Dependent variable: purchase intention. N = 383*,

* *p < 0.05*,

** *p < 0.01*,

+ *p < 0.1; The values in the parentheses represent Cronbach'α reliability coefficient*.

a *Gender (“0” male; “1” female)*.

b *Age (“1” under 18; “2” 18–25; “3” 26–30; “4” 31–40; “5” 41–50; “6” 51–60; “7” above 60)*.

c *Education (“1” high school and blow; “2” professional training; “3” bachelor; “4” master; “5” doctor and above)*.

However, from the perspective of data statistical analysis, we can only conclude that the interaction effect of an independent and a moderating variable is established through the hierarchical regression. To further investigate the moderating effect of the construal level, a bootstrapping analysis is necessary. By the bootstrap re-sampling method, statistical simulations can be performed based on traditional mathematical statistics. Based on this, the size of the moderating effect could be estimated. Subsequently, the estimated effect values are arranged from small to large, and the significance of the moderating effect value is estimated. When the CI of the estimated value of the moderating effect does not contain zero, the moderating effect is established.

Therefore, using Hayes's (2017) PROCESS macro (model 1; 5,000 bootstrapped samples), we conducted a moderation analysis. The bootstrapping further confirmed the second hypothesis (H2) of the moderating effect of construal level. The procedures generated a 95% CI around the moderating effect, with zero falling outside the CI under high-level construal (95% CI: 0.34–1.02; see the details in Table 4).

**Table 4:** Bootstrapping results for the moderating effect of construal level in Study 2.

	**Effect**	**SE**	**95% CIs**	
			**Lower limit**	**Upper limit**
Low construal level	−0.04	0.16	−0.34	0.27
Moderate construal level	0.29	0.11	0.07	0.51
High construal level	0.62	0.16	0.31	0.93

**Figure 3:**
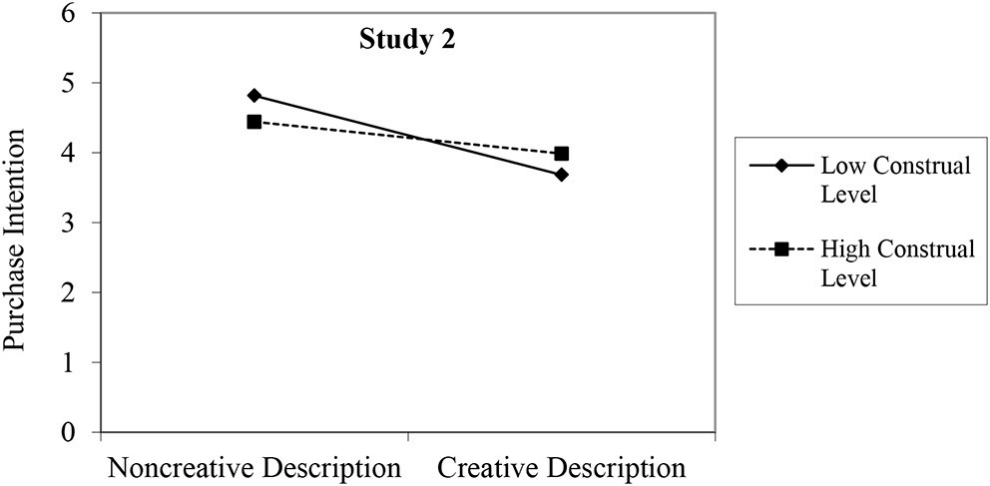
Moderating effect of construal level in Study 2.

Moreover, Study 2 conducted slope analyses to present the moderating effect of construal level (see Figure 3). We drew Figure 3 using Aiken and West's (1991) method; this figure indicates that the effects of a creative description on consumers' purchase intention for high-level construal consumers would be stronger than for low-level construal consumers.

#### Discussion

Upon replication of the results of Study 1, Study 2 provides additional support in favor of our framework. Especially, we found that construal levels as a personal trait could also play a similar role as a situation prime, which extends and enriches previous research (Landau et al., 2019). In summary, Studies 1 and 2 together provide solid empirical evidence for Hypotheses 1 and 2 by manipulating and directly measuring construal levels, respectively.

Since psychological distance is conceptually related to construal level (Baranan et al., 2006) as mentioned above, Study 3 would only manipulate construal level between the groups to further validate our core Hypothesis 2. Specifically, Study 3 will manipulate construal level through social distance (Goodman and Lim, 2018).

### Study 3

Study 3 aimed to further test our central hypothesis: the creative description's persuasiveness is stronger among high-level construal individuals. Similar to Study 1, Study 3 also primed construal level by a context cue. Between- and within-subjects mixed study design was employed in this study. In between-subjects, we manipulated construal level through social distance (Baskin et al., 2014; Goodman and Lim, 2018), which indicates being more or less distant in interpersonal closeness (Berscheid et al., 1989; Aron et al., 1992). In within-subjects, we exposed participants to both creative and non-creative descriptions of a product on two fictional e-commerce platforms and asked the participants to indicate their relative purchase preference between the two.

#### Method

##### Participants

We recruited 229 Chinese participants (83 males and 146 females) from WJX.cn for an online experiment, and they received a small monetary reward for their time. Most (68.1%) of them were aged 26–40 years. The sample sizes were based on *a priori* power analysis, and we sought a sample size, which is large enough to detect the mean effects reported in social psychology (*r* = 0.21 with 85% power at α = 0.05 (Richard et al., 2003).

##### Construal Level Manipulation and Manipulation Check

For priming construal level by a situational factor, Study 3 primarily followed Baskin et al.'s (2014) manipulation procedure and adopted the same manipulation check. The participants were randomly assigned to one condition in the two (distance: friend lives in the same town or more than 500 miles away) between-subjects designs at first. Borrowing from Baskin et al. (2014) and Goodman and Lim (2018), we asked the participants to think of a specific friend, either in their hometown or in a town at least 500 miles away, depending on their condition, and write the initials of that friend. Participants then spent at least 2 min writing about a time when they gave a gift to that friend. Then, similar to Baskin et al.'s (2014) research, participants completed an ostensibly unrelated task consisting of the BIF questionnaire as in Study 2 (α = 0.73; see the details in Table 1). This was used as a manipulation check for the construal level manipulation in this study (Baskin et al., 2014).

##### Shopping Scenario

Participants then entered an experimental shopping scenario that was also adapted from the procedure by Baskin et al. (2014). With the advancement of technology and people's pursuit of convenience, Bluetooth headphones are increasingly popular in recent years. Thus, participants were asked to imagine that they planned to buy Eros Bluetooth headphones as a present for the abovementioned friend, and that they were browsing the Eros store on two different e-commerce platforms: Atreus and Ladon. We assumed that these were trustworthy e-commerce sites that participants often shopped from. Participants were then presented with the pictures and text descriptions of Eros Bluetooth headphones from each platform; the pictures were the same, but the Atreus platform featured a creative text description and the Ladon platform featured a non-creative text description. Afterward, the participants were required to choose between the two e-commerce platforms and indicate their relative purchase intention on a 1–7 bipolar scale (Baskin et al., 2014; see the details in Table 1). The participants in the high-level construal condition were presented with the Atreus platform first, and their 1–7 bipolar scale was anchored at 1 = “prefer purchasing from Atreus” and 7 = “prefer purchasing on platform Ladon.” Low-level construal participants had a reversed. Finally, the participants gave their demographic information (gender, age, the level of education completed, etc.).

#### Results

**Figure 4:**
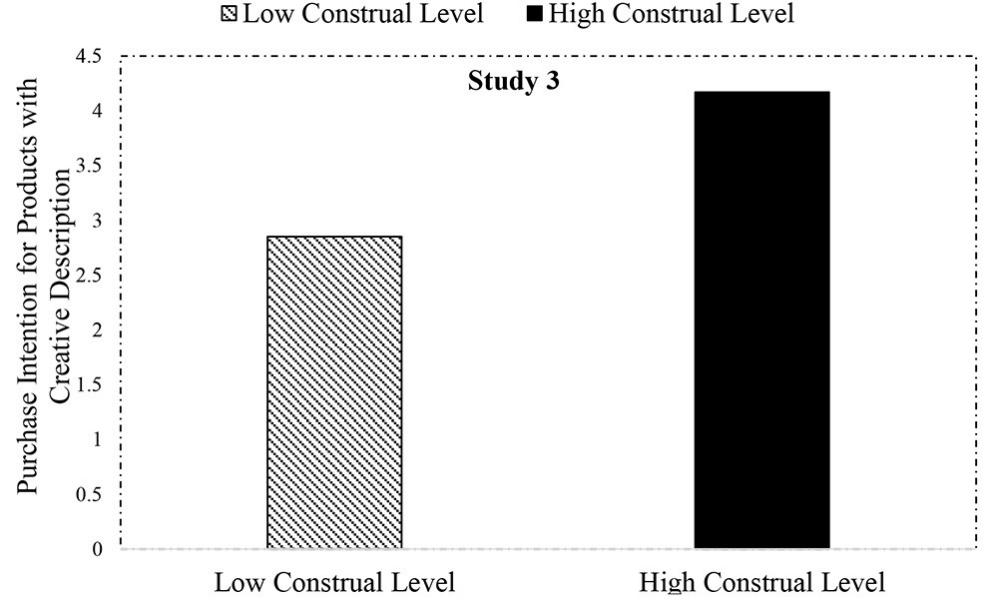
Effect of text description style and construal level on purchase intention in Study 3.

The manipulation of social distance indeed influenced the construal level scores [*M*_far_ = 17.88, SD = 4.58 vs. *M*_near_ = 12.83, SD = 2.31, *F*_(1, 227)_ = 113.47, *p* = 0.000, η^2^_*p*_ = 0.33]. Similar to Study 1, we conducted an ANOVA to compare the means of purchase preference between low- and high-level construal groups to test the moderating effect of construal level. As expected, the ANOVA using gender, age, and education as control variables revealed that construal level had a significant effect on the platform purchase preference. Specifically, high-level construal participants preferred the platform that featured a more creative description [*M*_far/purchasepreference_ = 4.17, SD = 1.84 vs. *M*_near/purchasepreference_ = 2.85, SD = 1.60, *F*_(1, 224)_ = 26.90, *p* = 0.000, η^2^_*p*_ = 0.11; see details in Figure 4], confirming H2.

#### Discussion

Differing from the previous two studies, Study 3 merely manipulates construal level between the groups with social distance, and the significant results provide additional support for our core Hypothesis 2: creative descriptions are highly persuasive to high-level construal individuals. This not only consolidates the framework of this research but also expands the relevant research on improving the effectiveness of creative marketing information, conforming to the call of West et al. (2019). Moreover, we provide new empirical evidence for the advantages of high-level construal in processing rhetorical devices (Shan et al., 2017; Landau et al., 2019).

Taken together, all three studies provide a convergent support for both of our hypotheses and robust results for our research model.

## General Discussion

This study proposed that the creative description positively affects consumers' purchase intention and proposed a boundary condition of construal level for this effect. Using three studies in manipulating or directly measuring construal level, we found that the creative text descriptions are generally more persuasive than the non-creative ones in an online context, and that the persuasiveness of the creative descriptions can be accentuated (vs. attenuated) especially for high- (vs. low-) level construal individuals. In the following sections, we have given in detail this paper's various theoretical and practical contributions.

### Theoretical Implications

This paper makes three main theoretical contributions. First, it demonstrates that construal level—that is, consumers' internal thoughts, thought processes, and interpretations, rather than external factors—influences its preference for a creative description style. Although most prior research on rhetorical advertisements examines several moderating factors, the perspectives primarily focus on the individual's motivation or ability (Phillips and McQuarrie, 2009; Chang and Yen, 2013). However, in the contemporary environment of multimedia and multitask, consumer's motivation or ability to process marketing communications is much lower than ever before (West et al., 2019; Zane et al., 2020). This indicates that those factors may not play their due role that scholars have found in traditional print advertisements. Moreover, prior research has put forward a process for rhetoric devices related to an individual's mindset, such as “top-down” thinking (Gibbs, 2013), cognitive flexibility (West et al., 2019), or even abstract thought (Vervaeke and Kennedy, 2004; Landau et al., 2019), which are all related to high construal level. Therefore, our results promote and integrate the relevant research on creative marketing messages and address various gaps in the literature.

Second, our research extends the applications of the construal level theory (Trope et al., 2007) to the field of creative marketing communications. Previous studies have suggested that consumers' construal level can systematically influence various aspects of consumer thinking, judgment, and behavior (Trope et al., 2007) and that the degree of match or fit between a persuasive message and consumers' mental representation of that message influences how persuasive it can be (Wheeler et al., 2005; Fujita et al., 2008). Though scholars have explored the role of an abstract mindset in metaphor communication (Landau et al., 2019), which is highly related to creative information, the role of construal level has not yet been explored in a broader creative marketing message field, even in an online context. The present study found that high-level construal strengthened creative descriptions' positive effects on consumer persuasion; therefore, it provides new and detailed evidence that construal level can influence the persuasiveness of a creative text description, which enriches the construal level literature.

Finally, by combining research on marketing communications using rhetoric devices and concentrating on the creative text descriptions in general, our claims integrate and extend other studies of creative marketing messages to the descriptions of products that appear online. This is a novel and necessary extension of the literature on traditional advertisements (Althuizen, 2017; Dahlén et al., 2018) given the prevalence of e-commerce today. This paper also identifies a moderator (i.e., construal level) that is more applicable to an online context, thus expanding and enriching the literature.

### Limitations and Future Research

This study has a few limitations. For instance, it focuses only on text descriptions of products. Future studies might take a more comprehensive look at how text descriptions combine with other elements of presentation, such as product images (including the use of anthropomorphism in advertising of brands Aggarwal and Mcgill, 2012; Wan et al., 2017) or the empty space surrounding the presentation (Kwan et al., 2017). Moreover, this paper only finds small effect sizes for the persuasiveness of creative product descriptions and merely examines construal level as a moderator of text description styles' divergent effects on purchase intention. Future studies might extend its findings by considering the combination of product description's creativity and other marketing factors, or by accounting for target demographics or the type of product for sale, or by examining various psychological mediators of text descriptions' styles by considering their prevalence online or consumers' penchant for online shopping.

### Practical Implications

This paper suggests that, to increase consumers' purchase intention, online retailers should take measures to increase consumers' construal level by enhancing psychological distance through presales or reminding consumers to buy the product as a present for others. Our findings also suggest that online retailers determine what their target customers already have so that they can maximize the effectiveness of figure descriptions; for instance, consumers habitually buy daily necessities such as food, etc. As the product selected in our three studies contained hedonic and utilitarian products, rather than necessary and unnecessary, we suppose, for daily necessities or food, consumers often have stocks or tend to habitually purchase (Vaughan, 2000), and whether to buy or not depends on the basic need. Therefore, the creative descriptions for food products may not be necessary or effective. Furthermore, we suggest that retailers create consumer profiles (Trusov et al., 2016), which might help them to target high-level construal consumers better and thus tailor their product's description toward those consumers. Since the effect sizes in our study are relatively small, it is also suggested that the creativity of product descriptions is one of many factors that affect consumers' purchasing decisions. Merchants should properly invest in it to cooperate with other marketing resources reasonably.

## Data Availability Statement

The raw data supporting the conclusions of this article will be made available by the authors, without undue reservation.

## Ethics Statement

Ethical review and approval was not required for the study on human participants in accordance with the local legislation and institutional requirements. Written informed consent from the participants' legal guardian/next of kin was not required to participate in this study in accordance with the national legislation and the institutional requirements.

## Author Contributions

F-SY and J-BS drafted and designed the work. F-SY and HZ collected the data. F-SY analyzed the data and drafted the manuscript. F-SY and J-BS critically revised the manuscript. All authors gave the final approval of the manuscript before the submission.

## Conflict of Interest

The authors declare that the research was conducted in the absence of any commercial or financial relationships that could be construed as a potential conflict of interest.
