# Supplementary material for: Is Creative Description Always Effective in Purchase Intention? The Construal Level Theory as a Moderating Effect
Source: Front Psychol. 2021 Jul 5;12:619340. doi: 10.3389/fpsyg.2021.619340 (PMC8287523; doi:10.3389/fpsyg.2021.619340)
Supplement: Supplementary file 1 [file Data_Sheet_1.docx]

Supplementary Material

# Marketing practices for the two description styles


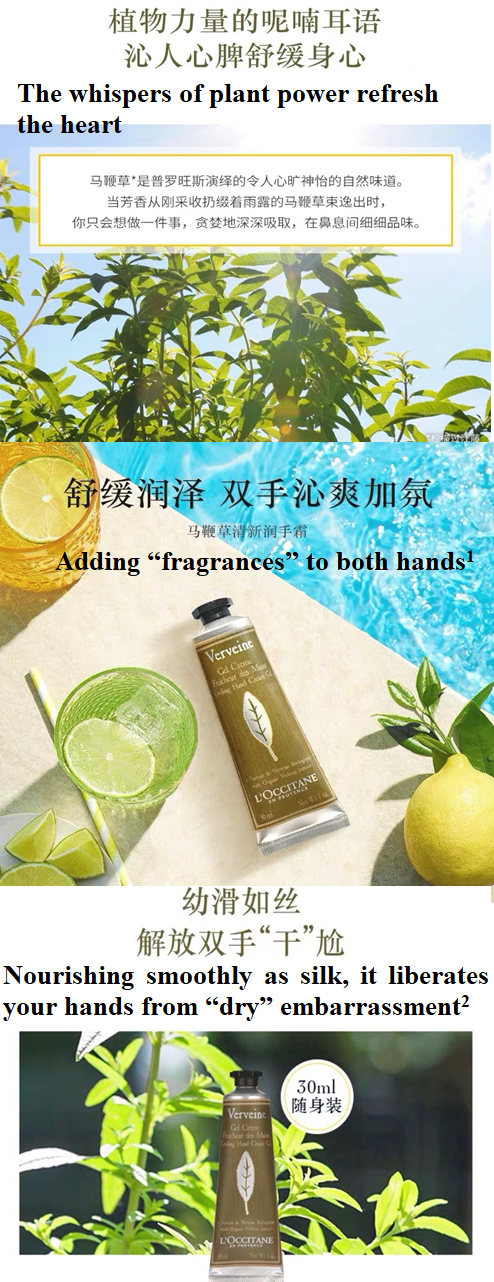


Figure 1. Creative Text Description

Note: The picture is originally in Chinese. The figurative text description comes from <https://detail.tmall.com/item.htm?id=42136409667&ut_sk=1.Xj/jvpmYXF0DAGOSuBmyIUDc_21380790_1590369353162.Copy.1&sourceType=item&price=90&suid=B7938B2F-C250-4A9F-83B3-73CD61912BDE&shareUniqueId=332919594&un=ab56d040636543983dd3af49e2902fce&share_crt_v=1&spm=a2159r.13376460.0.0&sp_tk=4oKzRkJpbjFKTnpaemjigrM=&cpp=1&shareurl=true&short_name=h.Vl0wGCm&sm=8892ae&app=chrome>. We provide a corresponding English translation for description section to help readers understand. The following contents are corresponding explanations for some words hard to present in English:

^1^ In the original Chinese version, “fragrances” is double quoted as “atmosphere” has the same pronunciation as “point,” both are pronounced “fēn.” Double quoting atmosphere means adding fragrance and point for you at the same time.

## ^2^ In the original Chinese version, “dry” is double quoted as “embarrassment” has the same first pronunciation as “dry,” both are pronounced “gān.” That means “‘dry’ embarrassment” and “embarrassment” are homophones.


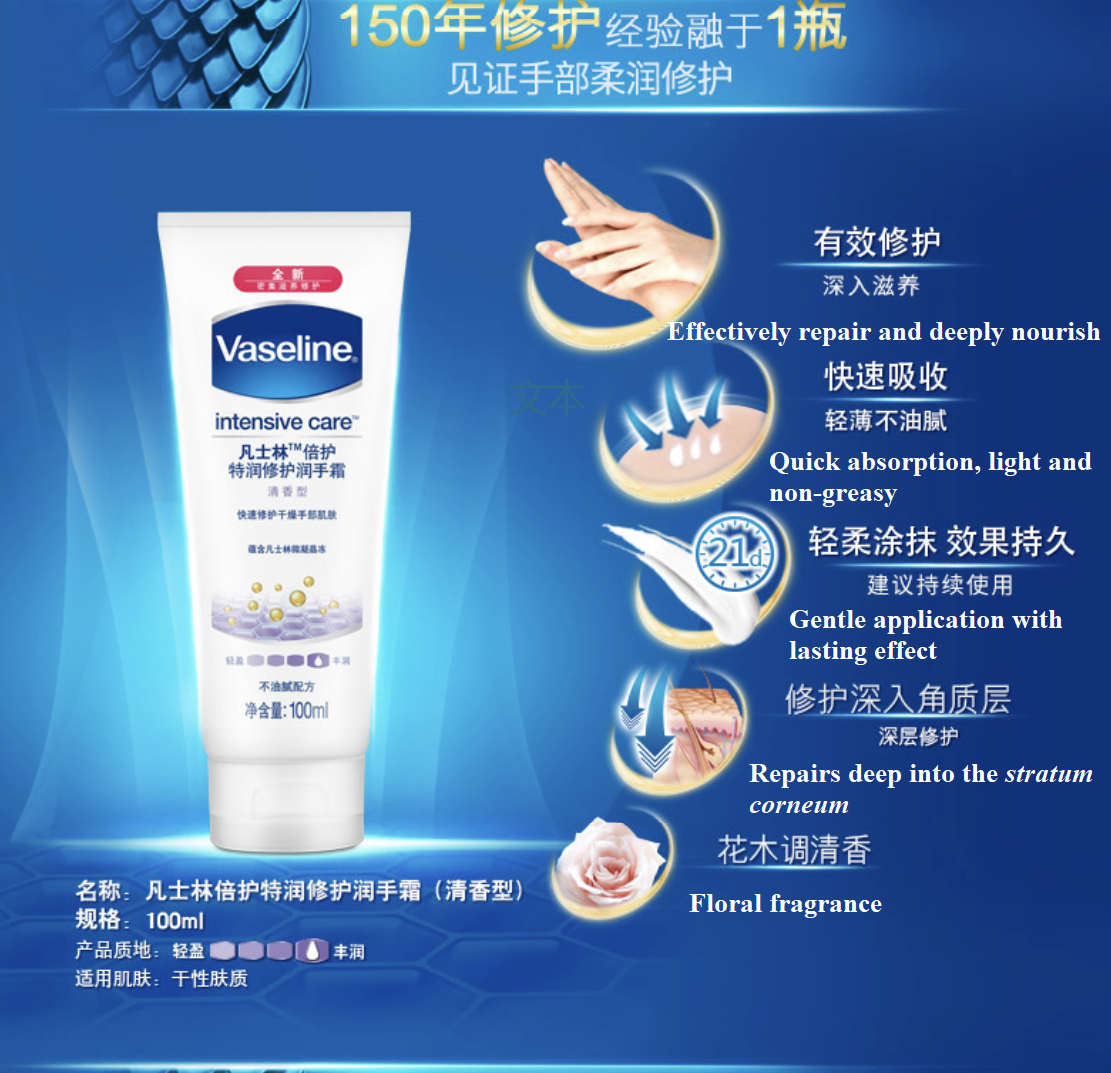


Figure 2. Noncreative Text Description

Note: The picture is originally in Chinese. The literal text description comes from <https://item.m.jd.com/product/11384079527.html?wxa_abtest=o&utm_source=iosapp&utm_medium=appshare&utm_campaign=t_335139774&utm_term=CopyURL&ad_od=share>. We provide a corresponding English translation for description section to help readers understand.

# Text descriptions and product pictures used in this research

| **Study ID** | **Creative** | **Noncreative** |
| --- | --- | --- |
| Study 1 | **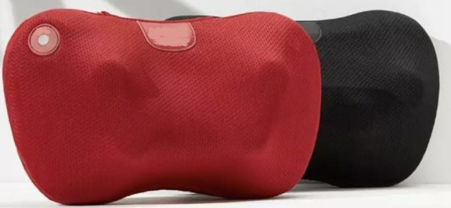**  **Intelligently manufactured^1^ by Juno, decompression massager**  - Start with the body and heal your fatigue.  - Butterfly wings shape firmly holds your body’s curve.  - Fist movement smoothly kneads every joint.  - Three-dimensional massage simulates the rhythm of a real-life massage.  - Strong torsion is skillfully applied to sore areas.  - Do whatever you want with the “body.”^2^  - One key, free control; one minute, automatic reversal.  - Automatically shuts down and protect you with actions.  - Vibrant red and elegant black, dazzling or classic, vivid or calm—you can choose. | **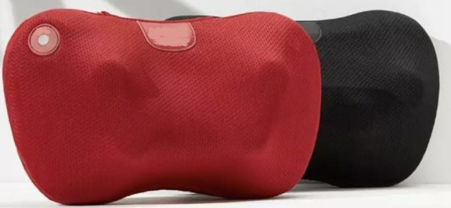**  **Made^1^ by Juno, decompression massager**  - Massage body to relieve fatigue.  - Thin arc design matches the curve of the human body.  - Flat massage smoothly kneads every joint.  - Three-dimensional massage simulates the rhythm of a real-life massage.  - Strong torsion is skillfully applied to sore areas.  - Suitable for many parts of your body.  - One button controls the start-up and switching massage modes; every minute, the rotation direction of the massage movement is automatically switched.  - Automatically shuts down without excessive massaging.  - There are two colors, red and black, and you can choose either one. |
| Study 2 | **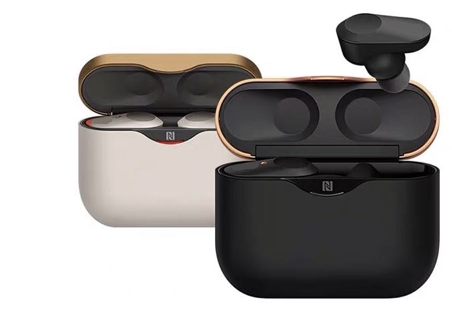**  **Listen to the pure and enjoy the quiet world, Eros wireless noise reduction Bluetooth headset 5.0**  - With these, you can say goodbye to headphone cables.  - Intelligent noise reduction, for quiet with environment.^3^  - Multiple functions, one finger control to open the intelligent “sound” life^4^.  - Comfortable silicone earplugs, your ears feel like they are touching a baby’s skin.  - The battery capacity should not be underestimated. With maximum battery life of about 32 hours and a fast charging function, it is always ready for you.  - The magnetic charging compartment can be fitted as soon as it is attached.  - Functional with no compromise in appearance with simple and stylish design. | **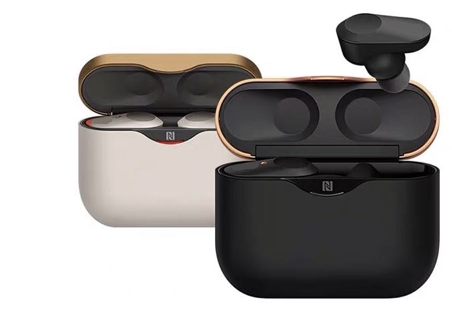**  **Eros wireless noise reduction Bluetooth headset 5.0**  - True wireless Bluetooth connection.  - Built-in noise reduction processor can achieve intelligent noise reduction.  - Turn on and off between songs with only one button.  - Comfortable silicone earplug design.  - Up to about 32 hours of battery life and fast charging function to meet all-day power needs.  - You can easily put the earphones in their magnetic charging box.  - Simple and stylish design. |
| Study 3 | **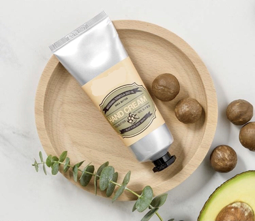**  **Hestia Verbena Shea Butter Hand Cream**  - Natural Verbena fragrance performed by Provence, the whispers of plant power refresh your hands and add “fragrances” to both hands.^5^  - Contains 16% of “female treasure”—shea butter. It liberates your hands from “dry” embarrassment.^6^  - Various capacity options and “hands” guard you anytime, anywhere.^7^ | **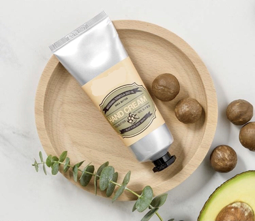**  **Hestia Verbena Shea Butter Hand Cream**  - Natural Provencal Verbena fragrance is extracted to moisturize your hands while adding fragrance.  - Contains 16% shea butter, which effectively repairs and nourishes dry hands.  - Various capacity options are available. |

Note: As all text descriptions are translated from Chinese. Some words are hard to present in English.

^1^ In the original Chinese version, “intelligently manufactured” and “made” are homophones; both are pronounced “zhì zào.”

^2^ In the original Chinese version, “body” is double quoted, as “Do whatever you want with the ‘body’” is adapted from the Chinese idiom, “Do whatever you want with the heart.”

^3^ In Chinese, “quiet” and “environment” are homophones. “Quiet” is pronounced “ān jìng” and “environment” is pronounced “huán jìng.” The original Chinese version chose their second pronunciations, which resemble one another.

^4^ In Chinese, “sound” is double quoted because “’sound’ life” and “life” are homophones. “Sound” in Chinese is pronounced “shēng yīn” and “life” is pronounced “shēng huó”. Both have the same first pronunciation as “shēng”.

^5^ In the original Chinese version, “fragrance” is double quoted as “atmosphere” has the same pronunciation as “point,” both are pronounced “fēn.” Double quoting atmosphere means adding fragrance and point for you at the same time.

^6^ In the original Chinese version, “dry” is double quoted as “embarrassment” has the same first pronunciation as “dry,” both are pronounced “gān.” That means “‘dry’ embarrassment” and “embarrassment” are homophones.

^7^ In the original Chinese version, “hands” is double quoted as “guard” has the same first pronunciation as “hands,” both are pronounced “shǒu.” That means “‘hands’ guard” and “guard” are homophones.

# Three pretests

Since more rhetoric devices in the creative text description and the description length between the more creative and less creative groups were not completely consistent. Therefore, we conducted three pretests for each study to remove some concerns.

The first purpose of pretests was to eliminate the concern about the cognitive load difference that may be caused by the inconsistent length between creative and noncreative product text descriptions. The longer one might cause more cognitive load (Paas, 1992). To eliminate the concern, we measured participant’s mental effort by a self-reported item with a scale (Paas, 1992) ranging from 1 (incredibly low mental effort) to 9 (incredibly high mental effort).

Second, though we have tried to merely control the creative group’s rhetoric devices while ensuring that the two descriptions’ core content was consistent, it was not easy to judge objectively whether the description content between the two groups was completely consistent. To rule out this concern, we ensured the pairs communicated the same message from the recipients’ perspective by asking them, “how well do you agree that the product description’s main message is...” according to the product’s different attributes (Dahlén et al., 2008).

Since Dahlén et al. (2008)’s research is aimed at creative advertisements and most advertisements have only one theme; thus, there is only one question in the original literature. However, the product description’s core content targeted by this research is the attributes of the product, and the difference in attributes determines the theme of each sentence. Therefore, in addition to individual attributes that can be combined into one question item, this research was based on the question item and formed the corresponding question item for each attribute description, respectively (see Table 1 for complete scales). We measured each item on a 7-point Likert scale anchored by 1 = not at all and 7 = very much (Dahlén et al., 2008).

The identification of each attribute in the product description was completed by three doctoral students who majored in marketing. First, each Ph.D. student individually read the product’s description of the two groups and wrote down each sentence’s product attributes. Subsequently, the three doctoral students jointly compared the product attributes they wrote down. Then, they negotiated and adjusted the differences until the three parties reached an agreement.

Last but not least, we also measured participant’s perceived creativity on three items (creative, interesting, novel; Madrigal and King, 2017) on a seven-point scale anchored by 1 = not at all and 7 = very much for each group to test whether the designed text descriptions were successfully manipulated.

Table 2. Items perception of the pairs communicated the same message used in post-tests

| **Construct (Source)** | **Post-test ID** | **Items** |
| --- | --- | --- |
| Perception of the pairs communicated the same message (Dahlén et al., 2008) | Pretest 1 | 1. How well do you agree that the above description text conveys the massager’s main function?  2. How well do you agree that the above description conveys the massager’s shape?  3. How well do you agree that the above description text conveys the massager’s rhythm and strength?  4. How well do you agree that the above description conveys the massager’s application part?  5. How well do you agree that the above description text conveys the massager’s convenient operation? 6. How well do you agree that the above description text conveys the massager’s optional colors? |
|  | Pretest 2 | 1. How well do you agree that the above description text conveys the wireless advantages of the Bluetooth headphones?  2. How well do you agree that the above description text conveys the noise reduction function of the Bluetooth headphones?  3. How well do you agree that the above description text conveys the convenient operation of the Bluetooth headphones’ buttons?  4. How well do you agree that the above description text conveys the comfort of the Bluetooth headphones’ silicone earplugs?  5. How well do you agree that the above description text conveys the standby time and charging convenience of the Bluetooth headphones’ buttons?  6. How well do you agree that the above description text conveys the Bluetooth headphones’ appearance? |
|  | Pretest 3 | 1. How well do you agree that the above description text conveys the natural addition and plant aroma of the hand cream?  2. How well do you agree that the above description text conveys the hand cream’s core ingredients?  3. How well do you agree that the above description text conveys the hand cream’s various specifications? |

## Pretest 1

Chinese participants (N = 204, 126 female) completed the study on WJX.cn in exchange for a monetary reward. The majority (65.2%) were aged 18 to 30 years old. The study employed a one factor (text description style: creative, noncreative) between-subjects design. The selected product was the same massager indicated in study 1.

Participants were randomly assigned to the two groups and read the same shopping scenario in study 1. After browsing, a questionnaire about mental effort, perception of the pairs communicated the same message (α = 0.79), perceived creativity of the text description (α = 0.77), and demographic variables was presented. All scales were shown in the above table and were translated into Chinese through the back-translation procedure, as all three studies did (Brislin, 1980).

Reassuringly, only perceived creativity differs significantly between the two groups. The creativity of text description perceived by the participants in the creativity group is significantly higher than that of the noncreative group (*M*_creative/perceived creativity_ = 5.36, SD = 0.83 vs. *M*_noncreative/perceived creativity_ = 4.91, SD = 1.07, *F*(1, 202) = 11.07, *p* = 0.001).

However, there were no significant differences of mental effort (*M*_creative/mental effort_ = 6.76, SD = 1.51 vs. *M*_noncreative/mental effort_ = 6.60, SD = 1.57, *F*(1, 202) = 0.59, *p* = 0.440) and perception of the pairs communicated the same message (*M*_creative/perceived same message_ = 5.41, SD = 0.79 vs. *M*_noncreative/ perceived same message_ = 5.31, SD = 0.82, *F*(1, 202) = 0.76, *p* = 0.383) between creative and noncreative text description.

## Pretest 2

Chinese participants (N = 204, 125 female) completed the study on WJX.cn in exchange for a monetary reward. The majority (69.6%) were aged 18 to 30 years old. The study employed a one factor (text description style: creative, noncreative) between-subjects design. The selected product was the Bluetooth headphones indicated in study 2.

Participants were randomly assigned to the two groups and read the same shopping scenario in study 2. After browsing, a questionnaire about mental effort, perception of the pairs communicated the same message (α = 0.81), perceived creativity of the text description (α = 0.71), and demographic variables was presented. All scales were shown in the above table and were translated into Chinese through the back-translation procedure, as all three studies did (Brislin, 1980).

Reassuringly, only perceived creativity differs significantly between the two groups. The creativity of text description perceived by the participants in the creativity group is significantly higher than that of the noncreative group (*M*_creative/perceived creativity_ = 5.34, SD = 0.75 vs. *M*_noncreative/perceived creativity_ = 5.02, SD = 1.07, *F*(1, 202) = 5.91, *p* = 0.016).

However, there were no significant differences of mental effort (*M*_creative/mental effort_ = 6.66, SD = 1.60 vs. *M*_noncreative/mental effort_ = 6.54, SD = 1.66, *F*(1, 202) = 0.29, *p* = 0.593) and perception of the pairs communicated the same message (*M*_creative/perceived same message_ = 5.42, SD = 0.84 vs. *M*_noncreative/ perceived same message_ = 5.26, SD = 0.82, *F*(1, 202) = 1.96, *p* = 0.163) between creative and noncreative text description.

## Pretest 3

Chinese participants (N = 149, 108 female) completed the study on WJX.cn in exchange for a monetary reward. The majority (67.1%) were aged 18 to 30 years old. The study employed a one factor (text description style: creative, noncreative) between-subjects design. The selected product was the hand cream indicated in study 3.

Participants were randomly assigned to the two groups and read the same shopping scenario in study 3. After browsing, a questionnaire about mental effort, perception of the pairs communicated the same message (α = 0.70), perceived creativity of the text description (α = 0.78), and demographic variables was presented. All scales were shown in the above table and were translated into Chinese through the back-translation procedure, as all three studies did (Brislin, 1980).

Reassuringly, only perceived creativity differs significantly between the two groups. The creativity of text description perceived by the participants in the creativity group is significantly higher than that of the noncreative group (*M*_creative/perceived creativity_ = 5.36, SD = 1.02 vs. *M*_noncreative/perceived creativity_ = 5.02, SD = 1.01, *F*(1, 147) = 4.10, *p* = 0.045).

However, there were no significant differences of mental effort (*M*_creative/mental effort_ = 6.57, SD = 1.76 vs. *M*_noncreative/mental effort_ = 6.39, SD = 1.80, *F*(1, 147) = 0.39, *p* = 0.532) and perception of the pairs communicated the same message (*M*_creative/perceived same message_ = 5.24, SD = 0.98 vs. *M*_noncreative/ perceived same message_ = 5.28, SD = 1.11, *F*(1, 147) = 0.05, *p* = 0.818) between creative and noncreative text description.

# Reference

Baskin, E., Wakslak, C. J., Trope, Y., and Novemsky, N. (2014). Why feasibility matters more to gift receivers than to givers: a construal-level approach to gift giving. *J. Consum. Res.* 41, 169-182. doi:10.1086/675737

Brislin, R. W. (1980). Translation and Content Analysis of Oral and Written Material. In H. C. Triandis (Ed.), Handbook of cross-cultural psychology (pp. 349-444). Boston: Allyn and Bacon.

Burgers, C., Konijn, E. A., Steen, G. J., and Iepsma, M. A. R. (2015). Making ads less complex, yet more creative and persuasive: the effects of conventional metaphors and irony in print advertising. *Int. J. Advert*. 34, 515-532. doi:10.1080/02650487.2014.996200

Dahlén, M., Rosengren, S., and Törn, F. (2008). Advertising creativity matters. *J. Advertising. Res*. 48, 392-403. doi:10.2501/S002184990808046X

Holzwarth, M., Janiszewski, C., and Neumann, M. M. (2006). The influence of avatars on online consumer shopping behavior. *J. Marketing*. 70, 19-36. doi:10.2307/30162112

Hong, J., and Lee, A. (2010). Feeling mixed but not torn: the moderating role of construal level in mixed emotions appeals. *J. Consum. Res*. 37, 456-472. doi:10.1086/653492

Madrigal, R., and King, J. (2017). Creative analogy as a means of articulating incongruent sponsorships. *J. Advertising.* 46, 521-535. doi:10.1080/00913367.2017.1396513

Paas, F. (1992). Training strategies for attaining transfer of problem-solving skill in statistics: A cognitive-load approach. *J. Educ. Psychol.* 84, 429-434. doi:10.1037/0022-0663.84.4.429

Vallacher, R. R., and Wegner, D. M. (1989). Levels of personal agency: individual variation in action identification. *J. Pers. Soc. Psychol.* 57, 660-671. doi:10.1515/cog-2015-0052

Visentin, M., Pizzi, G., and Pichierri, M. (2019). Fake news, real problems for brands: The impact of content truthfulness and source credibility on consumers’ behavioral intentions toward the advertised brands. *J. Interact. Mark.* 45, 99-112. doi:10.1016/j.intmar.2018.09.001
